# Metabolic traits in brown trout (*Salmo trutta*) vary in response to food restriction and intrinsic factors

**DOI:** 10.1093/conphys/coaa096

**Published:** 2020-10-14

**Authors:** Louise C Archer, Stephen A Hutton, Luke Harman, W Russell Poole, Patrick Gargan, Philip McGinnity, Thomas E Reed

**Affiliations:** 1 School of Biological, Earth and Environmental Sciences, University College Cork, Distillery Fields, North Mall, Cork T23 TK30, Ireland; 2 Environmental Research Institute, University College Cork, Lee Road, Cork T23 XE10, Ireland; 3 Marine Institute, Furnace, Newport, Co. Mayo F28 PF65, Ireland; 4 Inland Fisheries Ireland, 3044 Lake Drive, Citywest Business Campus, Dublin D24 Y265, Ireland

**Keywords:** anadromy, climate change, metabolism, partial migration, phenotypic flexibility, plasticity

## Abstract

Metabolic rates vary hugely within and between populations, yet we know relatively little about factors causing intraspecific variation. Since metabolic rate determines the energetic cost of life, uncovering these sources of variation is important to understand and forecast responses to environmental change. Moreover, few studies have examined factors causing intraspecific variation in metabolic flexibility. We explore how extrinsic environmental conditions and intrinsic factors contribute to variation in metabolic traits in brown trout, an iconic and polymorphic species that is threatened across much of its native range. We measured metabolic traits in offspring from two wild populations that naturally show life-history variation in migratory tactics (one anadromous, i.e. sea-migratory, one non-anadromous) that we reared under either optimal food or experimental conditions of long-term food restriction (lasting between 7 and 17 months). Both populations showed decreased standard metabolic rates (SMR—baseline energy requirements) under low food conditions. The anadromous population had higher maximum metabolic rate (MMR) than the non-anadromous population, and marginally higher SMR. The MMR difference was greater than SMR and consequently aerobic scope (AS) was higher in the anadromous population. MMR and AS were both higher in males than females. The anadromous population also had higher AS under low food compared to optimal food conditions, consistent with population-specific effects of food restriction on AS. Our results suggest different components of metabolic rate can vary in their response to environmental conditions, and according to intrinsic (population-background/sex) effects. Populations might further differ in their flexibility of metabolic traits, potentially due to intrinsic factors related to life history (e.g*.* migratory tactics). More comparisons of populations/individuals with divergent life histories will help to reveal this. Overall, our study suggests that incorporating an understanding of metabolic trait variation and flexibility and linking this to life history and demography will improve our ability to conserve populations experiencing global change.

## Teaser

Metabolism (rate of energy use) often varies considerably within species and populations. We found that food restriction (a potential outcome of environmental change) reduced baseline metabolism in brown trout, but traits also varied by sex and population factors. Understanding metabolic responses will help to predict/manage effects of global change.

## Introduction

Metabolic rate represents the fundamental energetic cost of living that underpins organism performance in variable environments. Since metabolism has important implications for fitness (Pettersen *et al.*, 2016, 2018), relatively higher or lower metabolic rates have been linked to variation in fitness components such as growth rates (Auer *et al.*, 2015c; Zeng *et al.*, 2017) and survival (Bochdansky *et al.*, 2005; Artacho and Nespolo, 2009) in ways that often depend on environmental context (Burton *et al.*, 2011; Auer *et al.*, 2015b, c). The minimum energy expenditure required for tissue maintenance and homeostasis is termed standard metabolic rate (SMR) in ectotherms [basal metabolic rate (BMR) in endotherms within the thermoneutral range]. SMR occurs when an organism is inactive, unstressed and non-digestive (Chabot *et al.*, 2016a). Maximum metabolic rate (MMR) sets the upper bounds of energy expenditure as the highest achievable rate of aerobic metabolism (transport of oxygen and production of Adenosine triphosphate) (Norin and Metcalfe, 2019). The difference between SMR and MMR defines an organism’s aerobic scope (AS), a trait that determines how much energy can be directed towards key functions including digestion, movement, growth and reproduction through increased metabolism, once baseline energy requirements (i.e. SMR) are met (Fry and Hart, 1948; Clark *et al.*, 2013).

Large variation in both SMR/BMR and MMR (and consequently AS) exists among species, populations, and individuals (Burton *et al.*, 2011; Konarzewski and Książek, 2013; Hillman *et al.*, 2013; Norin and Clark, 2016), with variation linked to differences in lifestyle (Killen *et al.*, 2010), geographic distribution (Angilletta, 2001; Naya and Bozinovic, 2012), thermal regime (Álvarez *et al.*, 2006; Eliason *et al.*, 2011; Sandblom *et al.*, 2016) and behaviour (Metcalfe *et al.*, 2016). In aquatic ectotherms, factors related to life-history tactics appear to underpin many inter-individual and intra-individual differences in metabolic traits (Metcalfe *et al*., 1995; McCarthy, 2000). Along with a 16-fold variation in MMR reported across fish species occupying different ecological niches (Norin and Clark, 2016), metabolic rates can still show *c*. 3-fold inter-individual variation after accounting for age and size (Metcalfe *et al.*, 2016). Such variation likely arises because the optimal combination of the various components of metabolic phenotype is context specific (Burton *et al*., 2011; Auer *et al.*, 2015b, c), or because populations (or types of individuals within populations) experience different selection pressures due to extrinsic or intrinsic factors, e.g. life-history differences/sex. For example, sockeye salmon *Oncorhynchus nerka* populations that undertake longer, or more challenging migrations have higher AS (Eliason *et al.*, 2011). Higher metabolic rates have also been documented in males versus females, e.g. higher AS in male pink salmon *O. gorbuscha* (Clark *et al.*, 2011).

Beyond variation per se, patterns of covariation in metabolic phenotypes can also differ across and within species. SMR and MMR are proposed to be tightly linked because of the ‘increased intake hypothesis’, whereby a high SMR requires investment in metabolic machinery that also facilitates a high MMR, with associated fitness benefits (Biro and Stamps, 2010; Burton *et al.*, 2011). While SMR and MMR generally do appear to be correlated within species (Auer *et al.*, 2017), the traits can vary in their response to different environmental factors, and the coupling of metabolic traits can be context dependent (Killen *et al.*, 2013; Norin *et al.*, 2016). Moreover, a decoupling of SMR and MMR can occur over time because each is under individual selection pressures (Norin and Metcalfe, 2019), which often operate in parallel but may also act independently (e.g. Wone *et al.*, 2015; Barceló *et al.*, 2016). Thus, even if SMR and MMR are somewhat functionally linked, ecologically significant variation in overarching AS can arise due to differences in the sensitivities of each metabolic trait to environmental conditions. Such within-individual variation in response to environmental variation may account to some extent for intraspecific patterns of variation and covariation in metabolic traits.

The ability of a single genotype to display different physiological, morphological or behavioural phenotypes in response to environmental variation is called phenotypic plasticity. Phenotypic ‘flexibility’ has been defined as a type of plasticity in which within-individual changes are reversible (Piersma and Drent, 2003), as distinct from developmental plasticity, where phenotypic responses to early developmental conditions remain relatively fixed for life (West-Eberhard, 2003). By facilitating individuals in coping with changing conditions (Seebacher *et al.*, 2015), phenotypic flexibility has life-history consequences that may scale up to affect higher levels of organization including population persistence, community stability and ecosystem processes (Bolnick *et al.*, 2011). Flexibility in metabolic rate is likely to be an important component here, with mounting evidence supporting metabolic plasticity as a widespread response to environmental change (Hofmann and Todgham, 2010). Factors including temperature (Seebacher *et al.*, 2015; Sandblom *et al.*, 2016; Morash *et al*., 2018), food availability (Auer *et al.*, 2015c, 2016a; Zeng *et al.*, 2018), food quality (Naya *et al.*, 2007), oxygen availability (Hochachka *et al.*, 1996; Norin *et al.*, 2016) and salinity (Allan *et al.*, 2006) can all induce short-term and longer-term (i.e. acclimation) changes in metabolic rates.

In ectotherms, SMR appears to be more flexible in the extent of its acclimation response to extrinsic factors than MMR (Norin and Metcalfe, 2019). For example, while acute exposure to warmer temperatures can result in elevated MMR (Guzzo *et al*., 2019), long-term increased temperatures caused European perch *Perca fluviatilis* to lower SMR, a thermal compensation response that was not apparent in MMR (Sandblom *et al.*, 2016). Greater flexibility in BMR relative to MMR (or cold-induced maximum aerobic metabolism) has been demonstrated in endotherms in response to temperature (Nespolo *et al.*, 2001; van de Ven *et al.*, 2013; Dubois *et al.*, 2016). Food availability can also induce flexibility in SMR (and BMR) (Naya *et al.*, 2007; Auer *et al.*, 2015c, 2016a; Langer *et al.* 2018), suggesting reductions in baseline metabolism, rather than MMR, tend to underpin overall metabolic flexibility in response to food restriction (Zeng *et al.*, 2018).

Although many studies report inter- and intra-specific variation in metabolic responses, we know considerably less about factors giving rise to differences in metabolic flexibility (Norin and Metcalfe, 2019). Variation in metabolic rate flexibility between populations has been described primarily as changes in SMR or BMR, and particularly in response to distribution or temperature factors, e.g. cane toads *Rhinela marina* at high latitudes show more plastic resting metabolic rate responses to temperature than their counterparts at low latitudes (Winwood-Smith *et al.*, 2015; McCann *et al.*, 2018). Similarly, rufous-collared sparrow *Zonotrichia capensis* populations from seasonally variable or temperate environments show more flexible BMRs in response to temperature than those from arid desert systems, though desert populations conversely showed more BMR flexibility at low food conditions, highlighting the context-dependency of optimal metabolic phenotypes (Cavieres and Sabat, 2008; Maldonado *et al.*, 2012). Since the optimal metabolic phenotype in a given context can vary considerably depending on population background, incorporating population-specific (or life-history) factors into the investigation of metabolic variation and flexibility is likely to have important implications for managing and conserving species experiencing environmental change, yet few studies have addressed this.

Salmonine fishes (salmons, trouts and charrs) represent an excellent group to study variation in metabolic phenotypes. As obligate freshwater spawning species, salmonines display a multitude of life-history strategies that often manifest as variation in migratory tactics (Klemetsen *et al.*, 2003). Some individuals remain resident in natal freshwaters for their entire life cycles, while others migrate to more productive feeding grounds such as larger rivers and lakes, or even undertake dramatic migrations to the sea (termed ‘anadromy’) (Ferguson *et al.*, 2019). Migration generally facilitates increased growth in the new habitat, with migrants typically returning to spawn in freshwater at larger sizes than non-migrants. Facultative migration—where individuals can adopt either a migratory or a non-migratory lifestyle—is common in salmonines, and populations can be primarily resident, migratory or comprise a mix of both tactics (Chapman *et al.*, 2012). Such alternative migratory phenotypes can be understood using the framework of the ‘environmentally cued threshold model’ (Tomkins and Hazel, 2007; Piche *et al.*, 2008; Pulido, 2011; Buoro *et al.*, 2012), whereby tactic frequencies are controlled by the relationship between an environmentally sensitive trait (e.g. physiological condition or energetic status) and a genetically variable threshold. Migration is triggered depending on whether or not an individual’s ‘status’ trait exceeds the threshold condition for residency. Energetic limitation in natal freshwaters is proposed to be a strong determinant of migration (Forseth *et al.*, 1999). As such, variation in migratory tactics is likely to be linked to variation in metabolic rates, e.g. steelhead trout *O. mykiss* that matured in freshwater without migrating tended to have lower SMR values (Sloat and Reeves, 2014) and juvenile Atlantic salmon *Salmo salar* with higher SMR early in life were more likely to subsequently migrate (McCarthy, 2000).

Once the migration decision has been made, different energetic demands are associated with each tactic. For example, migrants require sufficiently high aerobic capacity (i.e. MMR/AS) to sustain swimming performance during migration (covering distances of tens to several thousand kilometres) (Eliason *et al*., 2011) and to facilitate high growth in the new environment. In contrast, residents typically have lower energetic requirements, but must cope with fewer food resources in the freshwater environment (relative to marine resources) (Gross *et al.*, 1988). Populations displaying one predominant migratory phenotype are thus likely to experience different selection pressures on metabolic traits, whereby a migratory life-style might favour increases in the upper bounds of metabolisms (MMR), and residency might promote decreases in baseline energetic requirements (SMR), each with implications for overall AS and energy balance. This has some empirical support, for example, migratory three-spined stickleback *Gasterosteus aculeatus* had higher SMR, active metabolic rate and AS than non-migrants (Tudorache *et al.*, 2007; Dalziel *et al*., 2012a, b), and anadromous (sea-migratory) juvenile Atlantic salmon *S. salar* had higher SMR than non-migrants (Seppänen *et al.*, 2010). Less is known about how differences in migratory lifestyle might interact with environmental conditions to cause variation in metabolic traits.

Despite their economic importance and iconic status, many facultatively migratory salmonines are in widespread decline due to anthropogenic pressures including global change, in-stream barriers and the development of aquaculture (Limburg and Waldman, 2009). As traits of ecological relevance linking environmental conditions to individual performance and, ultimately, population dynamics (Seebacher and Franklin, 2012; Norin and Clark, 2016), understanding if and how metabolic traits respond to variable environmental conditions is crucial to successful conservation of threatened salmonine populations (Chabot *et al.*, 2016b). For example, while physiological plasticity generally confers resilience to environmental change (Seebacher *et al*., 2015), there are limits to metabolic compensation and many sockeye salmon *O. nerka* populations in the Fraser River—already operating close to their upper thermal limit of AS—are at risk from increasing water temperatures during migration (Eliason and Farrell, 2016).

Here, we explore the effects of extrinsic (food supply) versus intrinsic (population/sex) factors on metabolic rates in experimental F1 offspring derived from two wild populations of brown trout that differ in migratory tactics. Specifically, we aimed to (i) assess how long-term food restriction alters SMR, MMR and AS; (ii) test if population-background and sex influence variation in SMR, MMR and AS; and (iii) explore flexibility in metabolic traits with respect to food restriction. We expected that food restriction would have a greater effect on SMR compared to MMR. We also expected that offspring from the naturally migratory population would show relatively higher MMR or AS, and those from the non-migratory population would show relatively lower SMR.

## Materials and methods

### Study populations and fish rearing

Brown trout brood stock from two wild populations were caught by seine netting in November 2015 in the Erriff (National Salmonid Index Catchment) (53° 37´ N: 09° 40´ W) and Burrishoole (53° 57´ N: 09° 35´ W) catchments in the west of Ireland (Fig. S1). Erriff brood stock were caught in Tawnyard Lough, an upland lake of 56 ha which is fed primarily by the Glendavoch River and several smaller tributaries. The Tawnyard Lough population spawn mainly in the Glendavoch River, and move downstream as fry or parr to Tawnyard Lough (a distance of a few hundred metres to kilometres, depending on where spawning occurred). Tawnyard Lough produces a large run of out-migrating anadromous juveniles (smolts), with annual estimates of 500 to 3000 smolts enumerated at the outflow of the Lough over the past 30 years (Gargan *et al.*, 2016). An unknown proportion of the population remain within the lake, and undergo several years of freshwater growth before returning to the natal stream to spawn, with local expertise indicating that the Tawnyard population in general has a strong anadromous component (P. Gargan, *pers comm*).

Burrishoole brood stock were caught in Lough Bunaveela (46 ha) in the headwaters of the catchment. A population of non-anadromous trout remain resident in Lough Bunaveela for most of their lifecycle, undertaking only short-distance, directed movements (10–100 s of metres) between the lake and inflowing/outflowing spawning streams. Although the anadromous life history is present in the larger Burrishoole catchment, the development of aquaculture in Clew Bay is believed to have caused the anadromous trout run to decline severely in Burrishoole in the late 1980s. Despite Bunaveela spawning streams being accessible to anadromous fish, there is no evidence that the Bunaveela population has ever produced anadromous fish, either historically, or recently (Poole *et al.*, 2007; Magee, 2017). In summary, we consider offspring derived from the Tawnyard brood stock to have a strong anadromous background (hereafter termed the ‘anadromous background population’), and offspring from the Bunaveela brood stock to have no recent anadromous background (termed the ‘non-anadromous background population’).

See Archer *et al.*, (2019) for detailed description of crossing, fertilization and rearing procedures, described here in brief. Each ripe female was mated to two males from the same source population. Fertilized eggs were incubated at a hatchery in the Burrishoole catchment. Post-hatching, fry were transferred to a rearing facility at University College Cork (Aquaculture and Fisheries Development Centre). Here, fry were initially held in two 100 L growth tanks on a recirculating aquaculture system (RAS) maintained at temperatures typical of the west of Ireland, and moved to 520 L tanks on a larger RAS to facilitate growth in December 2016. Populations were reared separately throughout the study to prevent emergence of dominance hierarchies. Fry were fed *ad libitum* with commercially available trout pellets (Skretting Ltd, Norway) until experimental food treatments (see below) began in September 2016. During the experimental phase, a programmed lighting system of LED lights above each tank mimicked the photoperiod of the source catchments. Water was treated with mechanical filtration, bio filtration and UV skimming, and water quality (checked weekly) consistently remained within acceptable levels for fish health. Great care was taken to ensure that all measured variables other than feeding regime (fish densities, temperature, photoperiod, lux, flow rates) were constant across tanks.

### Food restriction treatments

Fish experienced experimental food restriction treatments from September 2016 to June 2018. The study, and all associated procedures, was carried out with ethical approval from Health Products Regulatory Authority Ireland, under project license AE19130/P034 and individual licenses AE19130/1087, AE19130/I200, AE19130/I201 and AE19130/I202 with all fish humanely euthanized under licence upon study completion.

To explore the effects of extrinsic environment (food restriction) and intrinsic factors on metabolism, juvenile brown trout from each population were randomly allocated one of four food treatments in September 2016 (*n* = 90 per feeding treatment per population, at the beginning of the experimental phase). The food treatments were: (i) High-High food: fish fed recommended daily pellet rations for optimal growth, calculated as a percentage of body mass and adjusted for seasonally changing temperatures (Skretting Ltd, Norway); (ii) Low-Low: fish fed 25% of recommended optimal rations; (iii) High-Low: fish switched from optimal rations to 25% optimal ration (i.e. from High to Low) in June 2017; and (iv) Low-High: fish switched from 25% of optimal rations to 100% optimal rations in June 2017 (i.e. from Low to High). The reductions to 25% of optimal food rations took place gradually over a 4-week period to minimize stress. Within each tank, absolute rations were adjusted monthly to account for changes in body mass and temperature.

### Measurement of metabolic traits

Eight to twelve individuals of each population in each food treatment were measured for SMR and MMR in February 2018 in a controlled-temperature (CT) chamber at 8°C (mean temperature 7.99°C ± 0.26 SD, matching the natural temperatures in the wild for these populations). Fish were kept at similar temperatures (mean = 7.49°C ± 1.56 SD) for ~1 month prior to respirometry.

### Measurement of MMR

Whole-animal oxygen consumption (*Ṁ*O_2_) in animals operating at their maximum aerobic metabolic rate was used as a proxy for MMR (Norin and Metcalfe, 2019) following best practices outlined in Norin and Clark (2016). We used an exhaustive chase protocol (Norin and Clark, 2016) to elicit MMR in the same individuals that we measured for SMR. Prior to SMR measurements, each individual fish was first placed in an aerated 50 L tank and manually chased by hand until exhaustion, determined to occur when the fish were unresponsive (i.e. did not elicit burst swimming) to tactile stimulus (typically after 2 to 3 min of sustained chasing). Once exhausted, the fish was immediately transferred to a respirometry chamber in the same system used to measure SMR, the chamber was sealed and oxygen decline within the closed chamber loop was recorded for a 60 s measurement period. The time taken to transfer fish to chambers and begin recording oxygen measurements never exceeded 20 s, ensuring minimal recovery from the exhaustive chase procedure occurred before measurements.

### Measurement of SMR

The SMR of individual fish was then determined overnight in a darkened CT chamber using intermittent-flow respirometry, following best practices outlined in Svendsen *et al.* (2016). The respirometry system comprised four acrylic respirometry chambers (1200 ml) (Loligo Systems, Viborg, Denmark), submerged in a water bath, flushed with de-chlorinated water bubbled to 100% oxygen saturation by an air stone. PVC tubing (10 mm diameter, non-permeable to oxygen) connected each chamber to two pumps (Eheim Ltd, Deizisau, Germany): the ‘flush’ pump flushed oxygenated water through the chambers. A second ‘recirculation’ pump recirculated water in a closed loop, whereby water exiting the chamber passed through a 10 mm flow-through oxygen cell (PreSens Ltd, Regensburg, Germany) before recirculating back. Oxygen level in each chamber was recorded at one second intervals to estimate oxygen decline in repeated cycles comprising a flush period (flush pump operational), and a measurement period (recirculation pump operational) when individual oxygen uptake (*Ṁ*O_2_, a proxy for SMR in fasted, inactive animals) was measured. Each cycle comprised 330 s of flushing, and a measurement period of 200–300 s (to ensure sufficient O_2_ depletion for calculating *Ṁ*O_2_ in different-sized fish). We allowed a 30 s buffer period before recording oxygen uptake in each measurement period, to allow the chamber water and flush water to mix completely and reach equilibrium oxygen saturation.

Fish were fasted for 28 h prior to respirometry measurements to ensure individuals were post-absorptive (Cutts *et al.*, 2002). Fish entered the chambers between 11:00 and 12:00 each day, and were left to acclimatize for ~5 h, with chambers continually flushed with oxygen-saturated water during this period. SMR measurements began between 15:00 and 16:00, and ended between 11:00 and 12:00 the following morning, allowing a minimum of 100 measurements of oxygen uptake per individual. Fish were not disturbed during this ~20-hr SMR measurement period. Once SMR measurements had finished, fish were removed from the chambers, lightly anesthetize with MS-222, blotted dry and mass and fork length were recorded. Each fish received an individual identifier tag using unique colour combinations of visible implant elastomer (Northwest Marine Technology Ltd, USA). To limit bacterial growth, the entire respirometry system was rinsed with bleach after each overnight SMR respirometry trial. We also measured background (i.e. bacterial) respiration in each chamber on a daily basis by recording oxygen decline in empty chambers for one measurement cycle before fish entered the chambers, and for one cycle after fish exited the chambers (post SMR measurements).

### Determination of sex

To determine sex, we euthanized fish that had been measured for MMR and SMR via an overdose of MS-222 in April 2018 [~2 months after respirometry measurements due to involvement in an ongoing parallel study, Archer *et al.* (2019)]. If sex could not be determined anatomically, genotypic sex was assigned using a microsatellite sex marker (P. Prodöhl, unpublished). We were unable to re-identify six individuals due to tag loss, leaving *n* = 55 fish successfully assigned for sex (28 females, 27 males). Sex ratios were similar across food treatment groups and across population backgrounds.

### Statistical analysis

To estimate SMR (mg O_2_ h^−1^), we first calculated *Ṁ*O_2_ values for each repeated measurement of oxygen uptake recorded during the overnight SMR respirometry trials. *Ṁ*O_2_ (mg O_2_ h^−1^) was calculated as the most consistent linear decline in oxygen recorded during each measurement cycle, estimated by rolling regression in the respR package in R (Harianto and Carey, 2019). All *Ṁ*O_2_ measurements were visually inspected to assess regression fit, and only *Ṁ*O_2_ values with an acceptable fit (associated R^2^ values > 0.90, unless a clear linear trend was determined upon visual inspection of fit) were included in subsequent SMR calculations. To account for any background respiration included in these *Ṁ*O_2_ values, we estimated background respiration by calculating *Ṁ*O_2_ values for the oxygen uptake measurements in empty chambers before and after each overnight SMR respirometry trial (as described above). Because background *Ṁ*O_2_ rates were assumed to increase linearly through time (due to bacterial growth), we allowed for a dynamic background correction value (i.e. that increased overnight), calculated as:

$$ \dot{M}{O}_{2\_ bg}={bg}_0+\left(t\times bg\right), $$

where *Ṁ*O_2_*bg*_ is background *Ṁ*O_2_, at a given measurement time point *t*, the time elapsed since initiating overnight SMR measurements, ${bg}_0$ and $bg$ are parameters (the intercept and slope, respectively) estimated from the matrix regression of background oxygen uptake before, and background oxygen uptake after, as a function of time elapsed. We then used *Ṁ*O_2_*bg*_ to account for background respiration by subtracting *Ṁ*O_2_*bg*_ from each value of *Ṁ*O_2_ as calculated for an individual fish at successive time points during the overnight SMR respirometry trials. *Ṁ*O_2_*bg*_ never exceeded more than 2% of total *Ṁ*O_2_ in all cases, indicating minimal background respiration.

SMR for each fish was calculated by taking the mean of the lowest 10th percentile of background-corrected *Ṁ*O_2_ values recorded over the 20-h SMR measurement period, then excluding outliers (values more than two standard deviations from this mean).

We estimated individual MMR (mg O_2_ h^−1^) using the respR package (Harianto and Carey, 2019) by calculating *Ṁ*O_2_ as the linear decline in oxygen in each chamber in the 60 s measurement period immediately after the exhaustive chase protocol (i.e. extracting slopes from the linear regression of oxygen concentration against time over a 60 s period). Oxygen sensor probe and equipment malfunctions resulted in respirometry measurements for six fish being discarded, leaving a total of *n* = 61 individuals measured for SMR and MMR. Absolute AS for each fish was calculated as MMR - SMR (mg O_2_ h^−1^).

Since metabolic responses to food restriction are well documented to be reversible in salmonines once standard food rations are reinstated (O’Connor *et al.*, 2000), we first assessed if any potential metabolic responses to food restriction had been reversed/offset in the Low-High treatments by February 2018 (when we measured metabolic traits). No differences in SMR (Analysis of variance: χ^2^ = 0.23,df = 1, *P* = 0.633), MMR (χ^2^ = 0.40, df = 1, *P* = 0.528)or AS (χ^2^ = 0.51, df = 1, *P* = 0.476) existed between the High-High and Low-High treatments. Similarly, we tested whether potential metabolic responses to food restriction were affected by the length of the food restriction period, i.e. did Low-Low (17 months restriction) differ from High-Low (7 months restriction). No differences existed in SMR (χ^2^ = 0.04, df = 1, *P* = 0.836), MMR (χ^2^ = 0.44, df = 1, *P* = 0.509) or AS (χ^2^ = 0.47, df = 1, *P* = 0.494) between the Low-Low and the High-Low treatments. Since our primary interest was simply in the overarching effects of food restriction on metabolism (and not the effects of switching food treatments per se), we combined the High-High and Low-High treatments into a single ‘High Food’ treatment group, and combined the Low-Low and the High-Low treatments into a single ‘Low Food’ treatment group. We present analyses using the ‘High Food’ and ‘Low Food’ groups here, with the caveat that ‘High’ or ‘Low’ refers specifically to the food treatment experienced in the ~7-month period prior to respirometry measurements [a timescale over which metabolic rates appear to be consistent in salmonines (Seppänen *et al.*, 2010)]. Moreover, pilot SMR measurements collected from our populations in April and May 2017 (following similar respirometry protocols to those described above) showed similar effects of High/Low food treatments as the results described below. This indicates that responses to food treatments were: (i) consistent though time (or at least between years) and (ii) most likely due to phenotypic plasticity rather than random variation.

To avoid the pitfalls associated with solely using *P*-values (Halsey *et al.*, 2015; Halsey, 2019), we tested for factors influencing mass-independent measures of SMR, MMR and AS through estimation statistics (estimating effect sizes) using the dabestr package (Ho *et al.*, 2019). We used the residuals of the linear relationships between log_10_ body mass, and SMR, MMR and AS (all log_10_ transformed) to correct for body size in these analyses. These residuals (rSMR, rMMR and rAS) gave mass-independent estimates of metabolic rates (individuals with positive residuals have higher than expected metabolic rates for a given mass, whereas negative residuals indicate lower than expected rates). Effect sizes for mean differences in rSMR, rMMR and rAS were computed for all pairwise comparisons between food treatments (high or low) and population background (anadromous or non-anadromous), and 95% confidence intervals (CIs) were constructed by bootstrapped resampling for 5000 resamples. We also tested for sex-based differences in metabolic traits by estimating effect sizes for pairwise comparisons of rSMR, rMMR and rAS between males and females. Analyses were also run using an alternative analysis of covariance approach, which tested for variation in the relationships between body mass and SMR, MMR and AS according to population, food treatment and sex using general linear models (GLMs) (see Supplementary material). These results (shown in the Supplementary material) were qualitatively similar, suggesting our findings based on estimation statistics are robust.

Finally, we explored whether population background and food treatments affected size-independent relationships between metabolic traits using GLMs (normal errors). One GLM included rMMR as a response variable, rSMR, food treatment and population treatment as explanatory variables, along with interactions between rSMR and food treatment, and between rSMR and population, and a three-way interaction term (rSMR × food × population). A second GLM included rAS as the response variable, and similarly included rSMR, food treatment, population treatment and interaction terms for rSMR × food, rSMR × population and rSMR × food × population. A third GLM included rAS as the response variable, and rMMR, food treatment, population treatment as predictors, along with interaction terms for rMMR × food, rMMR × population and rMMR × food × population.

For the estimation statistics, we considered an estimated difference in means between groups to exist (analogous to significance) if 95% CI of the effect size did not include zero. For GLM models, we used likelihood ratio tests to assess statistical significance of predictor variables at a 5% alpha level, with non-significant interaction terms excluded to interpret main effects. All models were checked against assumptions of the given model (independence, non-normality of residuals, heteroscedasticity and multicollinearity). Analysis was carried out in R version 3.6.0 (R Core Team, 2019).

## Results

### Effects of food restriction and population on metabolic rate

Whole-animal SMR, MMR and AS varied with food treatment and population (see Table 1 for mean values and SD by population and treatment combinations).

**Figure 1:**
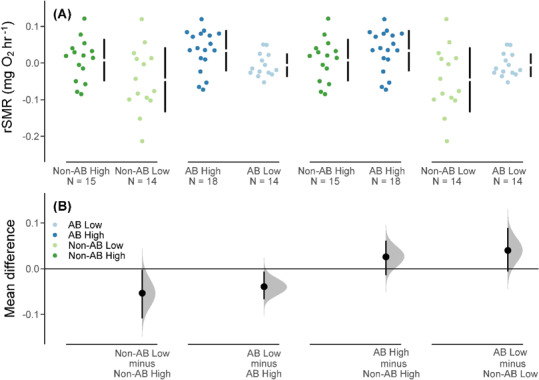
(**A**) Residual standard metabolic rate (rSMR) values (body mass corrected) for brown trout offspring derived from an anadromous background population (AB) and a non-anadromous background population (non-AB) that were reared under two experimental food treatments: optimal food rations (High) and 25% of optimal daily rations (Low) (black vertical bars represent the SD around the mean (shown as a gap in the bars), and sample size is shown as ‘N =’). (**B**) Cumming estimation plots for each population background and food treatment combination with effect sizes shown as black dots (i.e. the mean differences in rSMR among the groups), the distributions (shaded curves) and 95% CIs (back bars) of the effect sizes obtained from non-parametric bootstrap resampling (5000 resamples).

Fish from low food treatments had lower mass-independent SMR (rSMR) than those in the high food treatment (Fig. 1A), and this difference in mean rSMR was evident in both populations (Fig. 1A, Table 2). Fish from the anadromous background population had a marginally higher rSMR than those from the non-anadromous background population in both food treatments; however, the 95% CIs for the mean difference in rSMR between populations overlapped zero (Fig. 1B, Table 2).

**Table 1:** Mean values and associated standard deviations (SDs) for the length (mm), mass (g), standard metabolic rate (SMR) (mg O_2_ hr^−1^), maximum metabolic rate (MMR) (mg O_2_ hr^−1^) and AS (mg O_2_ hr^−1^) of brown trout offspring derived from two wild populations (AB = anadromous background population, non-AB = non anadromous background population)

**Food, Population**	**Length** (mean ± SD)	**Mass** (mean ± SD)	**SMR** (mean ± SD)	**MMR** (mean ± SD)	**AS** (mean ± SD)
High, AB	201.6 ± 18.8	109.65 ± 30.40	5.39 ± 1.71	40.52 ± 14.47	35.13 ± 13.38
Low, AB	200.6 ± 9.2	101.76 ± 16.38	4.51 ± 0.71	43.04 ± 8.94	38.52 ± 8.79
High, non-AB	199.7 ± 15.5	110.83 ± 27.59	5.18 ± 1.49	31.87 ± 10.79	26.69 ± 9.64
Low, non-AB	199.0 ± 17.7	101.37 ± 27.30	4.21 ± 1.54	30.31 ± 11.73	26.09 ± 11.13

Offspring were experimentally reared under two food treatments (High = optimal rations, Low = 25% of optimal rations)

**Figure 2:**
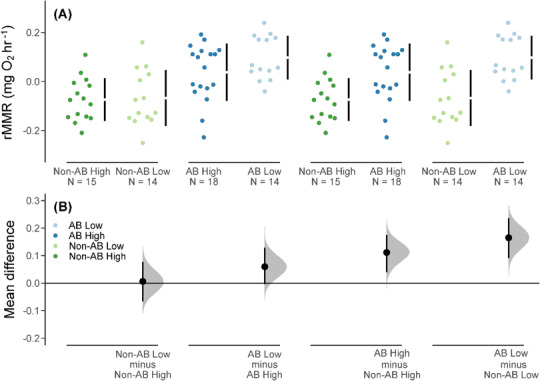
(**A**) rMMR values (body mass corrected) for brown trout offspring derived from an anadromous background population (AB) and a non-anadromous background population (non-AB) that were reared under two experimental food treatments: optimal food rations (High) and 25% of optimal daily rations (Low) (black vertical bars represent the SD around the mean (shown as a gap in the bars), and sample size is shown as ‘N =’). (**B**) Cumming estimation plots for each population background and food treatment combination with effect sizes shown as black dots (i.e. the mean differences in rMMR among the groups), the distributions (shaded curves) and 95% CIs (back bars) of the effect sizes obtained from non-parametric bootstrap resampling (5000 resamples).

There was no effect of food on rMMR in either population (95% CIs for the mean difference in rMMR overlapped zero, Fig. 2A and B). Fish from the anadromous background population had higher rMMR than those from the non-anadromous background population in both food treatments (Fig. 2A, B, Table 2).

**Table 2:** Effect sizes (∆) and associated 95% CIs for differences in mean residual standard metabolic rate (rSMR) (mg O_2_ hr^−1^), residual maximum metabolic rate (rMMR) (mg O_2_ hr^−1^) and residual aerobic scope (rAS) (mg O_2_ hr^−1^) of brown trout offspring derived from two wild populations (AB = anadromous background population, non-AB = non anadromous background population), exposed to two food treatments (High = optimal rations, Low = 25% of optimal rations)

**Mean difference (∆)**	**∆ rSMR (95% CI)** (*P*-value)	**∆ rMMR (95% CI)** (*P*-value)	**∆ rAS (95% CI)** (*P*-value)
*Low AB–High AB*	−0.039 (−0.067; −0.007) 0.024	0.060 (−0.003; 0.129) 0.129	0.075 (0.004; 0.155) 0.107
*Low non-AB–High non-AB*	−0.054 (−0.109; −0.002) 0.035	0.006 (−0.066; 0.078) 0.999	0.013 (−0.071; 0.101) 0.665
*High AB–High non-AB*	0.026 (−0.014; 0.061) 0.323	0.111 (0.039; 0.175) 0.006	0.125 (0.039; 0.200) 0.004
*Low AB–Low non-AB*	0.040 (−0.006; 0.089) 0.115	0.165 (0.091; 0.236) <0.001	0.187 (0.101; 0.271) 0.001
*Female–male*	−0.019 (−0.051; 0.020) 0.277	−0.155 (−0.203; –0.104) <0.001	−0.178 (−0.236; −0.118) <0.001

CIs were constructed by non-parametric bootstrap resampling (5000 resamples). Also shown for comparison are *P*-values of the two-sided permutation t-tests (5000 resamples)

**Figure 3:**
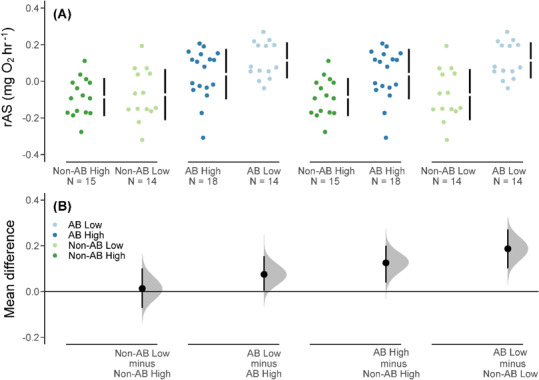
(**A**) rAS values (body mass corrected) for brown trout offspring derived from an anadromous background population (AB) and a non-anadromous background population (non-AB) that were reared under two experimental food treatments: optimal food rations (High) and 25% of optimal daily rations (Low) (black vertical bars represent the SD around the mean (shown as a gap in the bars), and sample size is shown as ‘N =’). (**B**) Cumming estimation plots for each population background and food treatment combination with effect sizes shown as black dots (i.e. the mean differences in rAS among the groups), the distributions (shaded curves) and 95% CIs (back bars) of the effect sizes obtained from non-parametric bootstrap resampling (5000 resamples).

Similarly, fish from the anadromous background population had a higher rAS than the non-anadromous background population under both food treatments (Fig. 3A and B, Table 2). We also detected population-specific effects of food treatment on rAS, where fish in the anadromous population in the low food treatment had a marginally higher rAS than those in the high food treatment (Fig. 3B, Table 2). This food treatment effect on rAS was absent in the non-anadromous population (Fig. 3B).

### Coupling of metabolic traits

**Figure 4:**
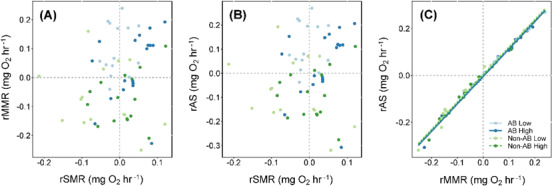
Size-independent relationships between: (**A**) residual standard metabolic rate (rSMR) and residual maximum metabolic rate (rMMR); (**B**) rSMR and rAS; and (**C**) rMMR and rAS for brown trout offspring derived from an anadromous background population (AB) and a non-anadromous background population (non-AB) that experienced two food reduction treatments: optimal food rations (High) and 25% of optimal rations (Low).

When considering effects of rSMR on rMMR (Fig. 4A), the interaction terms for rSMR × food × population (χ^2^ = 0.23, df = 1, *P* = 0.633), rSMR × food (χ^2^ = 1.45, df = 1, *P* = 0.229), rSMR × population (χ^2^ = 0.90, df = 1, *P* = 0.344) and food × population (χ^2^ = 2.66, df = 1, *P* = 0.103) were all non-significant. The main effects of rSMR (χ^2^ = 0.66, df = 1, *P* = 0.417) and food (χ^2^ = 2.21, df = 1, *P* = 0.137) were also non-significant. We detected a significant main effect of population background (χ^2^ = 22.35, df = 1, *P* < 0.001), whereby the anadromous background population had a higher rMMR for a given rSMR.

Effects of rSMR on rAS were similar (Fig. 4B). We detected non-significant interactions between rSMR × food × population (χ^2^ = 0.24, df = 1, *P* = 0.624), rSMR × food (χ^2^ = 1.39, df = 1, *P* = 0.239), rSMR × population (χ^2^ = 0.92, df = 1, *P* = 0.337) and food × population (χ^2^ = 2.69, df = 1, *P* = 0.101) and non-significant main effects of rSMR (χ^2^ = 0.004, df = 1, *P* = 0.952) and food (χ^2^ = 1.86, df = 1, *P* = 0.173). The anadromous population had a significantly higher rAS for a given rSMR (effect of population background: χ^2^ = 21.98, df = 1, *P* < 0.001).

We detected a significant positive relationship between rMMR and rAS (χ^2^ = 4689.8, df = 1, *P* < 0.001; Fig. 4C), but interactions between rMMR × food × population (χ^2^ = 1.16, df = 1, *P* = 0.201), rMMR × food (χ^2^ = 0.2, df = 1, *P* = 0.673), rMMR × population (χ^2^ = 2.3, df = 1, *P* = 0.1297) and food × population (χ^2^ = 0.1, df = 1, *P* = 0.768) were all non-significant. The main effects of food (χ^2^ = 2.4, df = 1, *P* = 0.123) and population (χ^2^ = 1.9, df = 1, *P* = 0.163) were also non-significant.

### Effects of sex on metabolism

**Figure 5:**
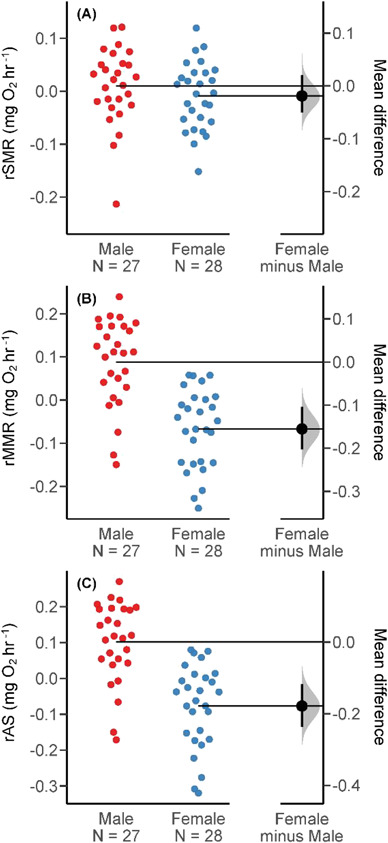
Gardner–Altman estimation plots for: (**A**) standard metabolic rate (SMR), (**B**) maximum metabolic rate (MMR) and (**C**) aerobic scope (AS) of male and female brown trout, which show the residual (body mass corrected) SMR/MMR/AS on the left axes and the effect size (mean difference between females and males) is represented by the black dot on the right axes, along with the distribution (shaded curve) and 95% CI (black bars) of the effect size, obtained via non-parametric bootstrap resampling (5000 resamples).

There were no sex-based differences detected in rSMR (Fig. 5A, Table 2). However, male fish had higher rMMR (Fig. 5B, Table 2) and rAS (Fig. 5C, Table 2) than female fish.

See Supplementary material for coefficient estimates for all of the above models.

## Discussion

Intraspecific variation in metabolic rates is widespread across species, yet there are still gaps in our understanding of how intrinsic and extrinsic environmental factors can collectively influence the various components of an individual’s metabolism. Here, we exposed brown trout offspring from divergent population backgrounds (one anadromous, one non-anadromous) to long-term food restriction to determine if, and how, extrinsic factors (food resources) versus intrinsic factors (population/sex) affect metabolic rates. Low food conditions resulted in a lower SMR with slight differences in overall SMR between populations. Fish from the anadromous population had higher MMR, and consequently, higher AS than the non-anadromous population under all food regimes. Intriguingly, fish from the anadromous background also had higher AS at low food compared to high food conditions, suggesting this population was more flexible in AS than the non-anadromous population. We also found sex-based differences in MMR and AS, which were not apparent in SMR. Collectively, our results suggest the various components of metabolism are differentially affected by intrinsic and extrinsic factors. Moreover, populations may vary in their capacity to flexibly adjust metabolic traits in response to environmental conditions, with consequences for population resilience to global change.

### Effects of extrinsic environment on metabolic traits

The lower SMR we observed in response to long-term food restriction is in line with previous work showing SMR (or BMR) to be strongly sensitive to food availability, typically without corresponding changes in MMR (Metcalfe *et al.*, 2016). SMR has shown similar flexible decreases in food-poor environments (Naya *et al.*, 2007; Auer *et al.*, 2015c, 2016a; Zeng *et al.*, 2018; Langer *et al.*, 2018) or increases at high food availability (Van Leeuwen *et al.*, 2011, 2012). Reductions in SMR are assumed to be optimal when food is scarce because the overall energetic cost of living is similarly reduced, thus facilitating higher growth (and consequently, fitness) (Auer *et al.*, 2015c), while a higher SMR is preferable (under the increased intake hypothesis) when environmental conditions support growth (Biro and Stamps, 2010; Burton *et al.*, 2011). Such flexibility in SMR will likely have positive implications for species experiencing rapid environmental change, with temperature-induced plasticity in SMR linked to increased resilience to climate change (Magozzi and Calosi, 2015). However, the adaptiveness of a given flexible response will depend upon both the predictability of the environmental change (i.e. the pattern of fluctuations in the environment), and the speed at which organisms can flexibly adjust their phenotypes to match these changing conditions (Reed *et al.*, 2010). Moreover, it is unclear whether SMR flexibility translates into overall fitness benefits in scenarios of multi-faceted environmental change. This is particularly pertinent for aquatic ectotherms such as salmonines, which are likely to experience reductions in invertebrate prey size and abundance alongside warming (Durance and Ormerod, 2007). It remains to be seen whether such populations have the capacity to sufficiently reduce SMR in response to combined stressors of food restriction and warming, though a study in common carp *Cyprinus carpio* indicates that the benefits of food-induced SMR plasticity may be temperature dependent (Zeng *et al.*, 2018).

### Effects of intrinsic factors on metabolic traits

As expected, we detected overall variation in SMR, MMR and AS according to population, with higher metabolic traits observed in the anadromous population. Population-level variation in metabolic traits could either arise through plasticity/flexibility/acclimation, or reflect genetic differences (which could include genetic variation in plasticity itself, e.g. variation among genotypes in their extent of flexibility). Since we observed population-level differences in metabolic traits in both high and low food treatments, differences between populations are more likely due to inherited genetic differences rather than plastic responses [i.e. the non-anadromous population had lower MMR and AS, and a marginally lower (though non-significant) SMR even at optimal food levels]. Metabolic rates are evolvable (Pettersen *et al.*, 2018) and respond to selection across relatively short time frames, e.g. BMR increased within 11 generations of selection in bank voles *Myodes glareolus* (Sadowska *et al.*, 2015). Such inherited differences could arise from standard inherited allelic variation, or from inherited environmental influences (e.g. maternal effects) that could include epigenetic inheritance. Regardless of the inheritance mechanism, the resulting fixed phenotypic differences could be adaptive, and perhaps indicative of life-history differences between the populations. For example, the non-anadromous population may have experienced stronger selection for reduced SMR in order to minimize their baseline energy requirements in freshwater habitats with lower prey resources (Gross *et al.*, 1988), whereas selection on SMR may have been in the opposite direction in the anadromous population, whereby higher SMR (and indeed MMR or AS) could facilitate rapid somatic growth in order to reach target smolt sizes to successfully migrate (McCarthy, 2000). Moreover, the decoupling of SMR and MMR we detected suggests that these metabolic traits are under subtly different selection pressures (Norin and Metcalfe, 2019). We acknowledge, however, that here we consider one anadromous and one non-anadromous population, and that further work with incorporating more populations (or individual-level data that links metabolic phenotypes to life histories) is required to generalize these findings. The results are nonetheless in line with expectations based on salmonid biology and the broader literature.

While the effect size of population on SMR was small in both food treatments (95% CIs included zero, suggesting marginal population-level differences), the relatively strong population differences in MMR and AS we observed suggests the upper bounds of metabolism may be more affected by intrinsic (i.e. genetic/evolved) factors than the lower bounds. As we expected, the anadromous population showed comparatively higher MMR than the non-anadromous population, a finding that could potentially reflect differences in migratory life histories, e.g. MMR and AS differences have been previously described in migratory versus non-migratory ecotypes of three-spined sticklebacks (Tudorache *et al.*, 2007). A genetic basis to MMR has been proposed to underpin metabolic variation between migratory forms of three-spined stickleback, where differences in MMR between anadromous and non-anadromous populations have been explained within the context of relaxed selection on swimming performance in stream-resident populations, mediated by reductions in MMR (Dalziel *et al.*, 2012b). In contrast, anadromous populations that undertake more arduous/lengthy migrations tend to have higher swimming/cardiac performances, and higher MMR (Lee *et al.*, 2003; Eliason *et al.*, 2011; Dalziel *et al.*, 2012a), indicating migration effort could further underpin consistent differences in MMR between migratory and non-migratory individuals or populations (Seppänen *et al.*, 2010). A higher MMR in the anadromous population may also confer fitness benefits by facilitating high levels of growth through direct selection on MMR in the freshwater environment (where fast growth increased migration success). Indeed, differences in intrinsic freshwater growth rates have been described for our study populations (Archer *et al.*, 2019). Indirect selection on MMR in juveniles might also occur because of a positive genetic correlation with MMR expressed in the marine environment, where high growth rates are translated into increased fecundity, with rank-order MMR in fish generally repeatable through time (Norin and Clark, 2016). Future comparisons of more populations/individuals with divergent life histories will shed more light on this.

Sex-based differences in MMR (and AS) that were not evident in SMR further suggest that MMR is more strongly influenced by intrinsic rather than environmental factors. We observed higher MMR in males, suggesting that despite similar basic energetic requirements in both sexes, males had more scope to increase their metabolism and divert resources into processes such as growth, or aggressive interactions underpinning competition. Salmonines generally show patterns of sex-specific aggression, with differences in aggression developing early, e.g. juvenile *O. mykiss* display more aggressive behaviour than females (Johnsson and Åkerman, 1998), a trait likely genetically correlated to sex-based differences in competitive ability as adults (Johnsson *et al.*, 2001). On a broader scale, our finding corroborates evidence for sex-specific differences in AS described in pink salmon *O. gorbuscha* (Clark *et al.*, 2011), and in cardiovascular performance of migrating sockeye salmon *O. nerka* (Sandblom *et al.* 2009). Collectively, these studies suggest that the relatively lower AS of female salmonines could make them more susceptible to global change.

### Differences in flexibility of metabolic traits

That we detected stronger effects of food restriction on SMR compared to MMR or AS lends further support to suggestions that the ‘ceiling’ (MMR), which constrains upper limits of metabolism, is less flexible than the metabolic ‘floor’ (SMR) (Sandblom *et al.*, 2016). Nonetheless, we detected population-specific flexibility in AS, where the anadromous population had marginally higher AS at low rather than high food conditions. The AS flexibility in the anadromous population appeared to be somewhat underpinned by decreased SMR at low food conditions (i.e. similar effect sizes for food treatment effects on SMR in both populations, but higher/positive effects sizes of low food on AS were only seen in the anadromous population). The few studies that have explored food restriction effects on MMR or AS have found little evidence for food-induced flexibility in these traits (Van Leeuwen *et al.*, 2011; Killen, 2014; Auer *et al.*, 2016b; Zeng *et al.*, 2018). The observed increase in AS under food restriction is initially counter-intuitive, but can be interpreted as further evidence for the optimal combination of metabolic traits being context-dependent*.* For example, context-dependency of flexibility in MMR and AS have been described in barramundi *Lates calcarifer* that showed variable plasticity to hypoxia, salinity and temperature changes (Norin *et al.*, 2016). It is less clear why a higher AS might be optimal in a low food environment. The ability to flexibly increase AS may perhaps be a consequence of the migratory background of this population, particularly if the conditions that promote a migratory life history in this population also tend to promote flexibility in SMR, MMR or AS (e.g*.* fluctuations in food resources/quality in the catchment-of-origin—or in the migratory destination—could drive patterns of migration and also flexibility in AS).

Plasticity in AS in the anadromous population could also be an adaptive response to conditions of low food, given that food restriction increases the frequency of migration in brown trout (Ferguson *et al.*, 2019 and references therein) and has been shown to increase the prevalence of migrants in this population (Archer *et al.*, 2019, 2020). If low food environments promote migration, individuals that can increase AS might have higher fitness, since high aerobic capacity (i.e. MMR or AS) is required to fuel swimming performance of migrating fish (Claireaux *et al.*, 2005; Eliason *et al.*, 2011). A flexible AS in low food conditions could thus be a population-level adaptive response to a migratory background, a response that only emerges under food limitation when it potentially facilitates improved migration performance. Such a response is comparable to documented increased oxygen consumption of high latitude (i.e. cold-acclimated) killifish *Fundulus heteroclitus* compared to low-latitude fish, with differences only evident at cold extremes (Fangue *et al.*, 2009; Dhillon and Schulte, 2011). More broadly, if environmental factors such as low food or inclement temperatures interact with metabolic traits to alter the costs and benefits of life-history tactics (e.g. migration versus residency tactics), an intriguing interplay between environmental and evolutionary processes could emerge. A tight evolutionary coupling has recently been described for metabolic rate and pace of life history in guppies *Poecilia reticulata* (Auer *et al.*, 2018b), and similar mechanisms could operate in species with alternative migratory tactics.

Higher AS (but not SMR) has been linked with competitive performance (Killen *et al.*, 2014) and increased food intake (Auer *et al.*, 2015a). Selection for higher MMR in low food environments has recently been shown in juvenile Atlantic salmon *S. salar* (Auer *et al.*, 2018a), with higher MMR explained in relation to increased competitive ability. As such, a flexible MMR may represent an alternative metabolic strategy to lowering SMR in order to maintain food consumption rates (and growth) in response to long-term food restriction, e.g*.* increased MMR (translating into higher AS) might allow for increased foraging/search effort, or enhanced competitive ability when food is limited (Auer *et al*., 2018a). This alternative metabolic strategy could be beneficial in the context of long-term food restriction scenarios because SMR depression is associated with the accumulation of harmful mitochondrial reactive oxygen species that can impose long-term costs on life-history traits (Salin *et al.*, 2018). Alternatively, we cannot rule out that food reduction may have induced higher MMR as a by-product of increased numbers of aggressive interactions between individuals (Seebacher *et al.*, 2013), with anadromous brown trout tending to show more aggressive behaviour than non-anadromous forms (Lahti *et al.*, 2001).

### Implications and considerations

It is important to note that we only considered metabolic variation at a single life stage, and it is likely that metabolism (and its flexibility) can vary though ontogeny (Pettersen *et al.*, 2016), depending on energy requirements associated with developmental stage (Beaman *et al.*, 2016; Burggren, 2018), or seasonal changes (Versteegh *et al.*, 2012; Petit *et al.*, 2013). Expanding our approach to incorporate repeated measurements of individuals throughout their lives (or individuals at different stages of ontogeny) would further illuminate when and how metabolic flexibility develops, and is most beneficial. While the importance of population background for traits related to metabolism is clear, it is less obvious whether such population-background effects can be attributed to life-history differences between populations, or are simply indicative of the different catchments of origin (i.e. the various populations having evolved in distinct river systems, possibly originating from different lineages). Inclusion of additional populations of divergent life histories would help to parse out factors related specifically to life history from those related to various other population-level differences. Nevertheless, life history is intricately linked to population-specific factors, being both proximately, and ultimately (via selective forces) determined by such factors (e.g. growth opportunity in the local environment), and is thus likely to be representative of any major differences between populations.

Collectively, our results indicate that metabolic traits can respond differently to extrinsic and intrinsic factors, and metabolic responses can further vary according to intrinsic (population-specific) factors. Developing an improved understanding of interactions between extrinsic and intrinsic factors, as mediated via metabolic physiology, will improve our ability to anticipate population responses to environmental change. That maximum metabolism was more fixed than minimum metabolism suggests that trout may be more constrained in their capacity to adjust AS in response to extrinsic factors, potentially making them more vulnerable to factors associated with global change [e.g. increased temperatures, changes in food supply and habitat degradation (Durance and Ormerod, 2007; Crozier *et al.*, 2008; Limburg and Waldman, 2009)]. This is particularly relevant for populations facing threats stemming from environmental change (e.g. in southern Europe, Almodóvar *et al*., 2012) but also has important general conservation implications given the species is already in decline across much of its native range due to anthropogenic activities (Limburg and Waldman, 2009). Moreover, the observed variation in metabolic traits according to intrinsic factors indicates that responses to environmental change are unlikely to be universal, making development of effective management strategies more complex. Nevertheless, greater plasticity is linked to higher resilience (Magozzi and Calosi, 2015; Seebacher *et al.*, 2015) if environmental changes are predictable (Reed *et al.*, 2010) and understanding the capacity of species and populations to flexibly adjust their metabolic traits is essential for predicting and mitigating the effects of progressively changing environmental conditions in natural systems.

## Contributions

L.A., T.R. and P.McG. conceived the study. L.A., S.H., P.G. and L.H. collected data and contributed to experimental design. L.A. conducted statistical analysis and wrote the manuscript. All authors contributed to interpretation of results and revisions of the manuscript.

## Data availability

The data supporting the results of this study will be made available upon reasonable request from the corresponding author.
